# IDO Inhibitor and Gallic Acid Cross-Linked Small Molecule Drug Synergistic Treatment of Melanoma

**DOI:** 10.3389/fonc.2022.904229

**Published:** 2022-07-01

**Authors:** Hongmei Liu, Huan Gao, Cheng Chen, Wenyu Jia, Delong Xu, Guan Jiang

**Affiliations:** ^1^ Xuzhou Medical University, Xuzhou, China; ^2^ Department of Biomedical Engineering, Southern University of Science and Technology, Shenzhen, China; ^3^ Department of Dermatology, Affiliated Hospital of Xuzhou Medical University, Xuzhou, China

**Keywords:** gallic acid, indoleamine 2, 3-dioxygenase, melanoma, chemotherapy, immunotherapy

## Abstract

In this study, we synthesized a molecule GA-1MT (GM) composed of indoleamine 2,3-dioxygenase (IDO) inhibitor (1-methyl-d-tryptophan, 1MT) called NLG8189 and gallic acid (GA) and verified its therapeutic effect on B16F10 melanoma cells and an orthotopic tumor-bearing mouse model. The synthesized molecule GM was analyzed by ^1^H NMR and mass spectrometry (MS). In addition, we confirmed that GM could mediate the immune response in the B16F10 cell tumor model by flow cytometry and immunofluorescence. The synthesized GM molecule could increase the solubility of 1MT to enhance the drug efficacy and lower costs. Moreover, GM could inhibit melanoma growth by combining 1MT and GA. *In vivo* experiments showed that GM could effectively inhibit the expression of tyrosinase, regulate the proportion of CD4^+^ T cells, CD8^+^ T cells, and regulatory T cells (T_reg_ cells) in tumors, and significantly suppress melanoma growth. The newly synthesized drug GM could more effectively inhibit melanoma than GA and 1MT alone or in combination.

## Introduction

Melanoma is an extremely malignant cancer that accounts for less than 20% of all skin cancers (1) but accounts for more than 80% of deaths caused by skin cancers (2). Melanoma often exhibits high invasion ability and metastasizes to the brain, liver, lung, and skin. Current evidence suggests that conventional chemotherapy has little effect on melanoma (2). The immunotherapy drug called 1-methyl-d-tryptophan (1MT) is well established to have poor water solubility, which restricts its clinical treatment for the treatment of melanoma (3, 4). Chemotherapy combined with immunotherapy was proved to enhance the efficacy of drugs (5). Bourquin et al. confirmed that chemotherapy combined with immunotherapy could enhance the efficacy of chemotherapy drugs and achieve a multiplier effect (6). Gallic acid (GA) is a natural polyphenol that can be extracted from various plants (7). Studies have found that natural polyphenols can inhibit tumor progression through various mechanisms, such as inhibiting tumor proliferation and migration and inducing apoptosis (8–10). It can also decrease the activity of matrix metalloproteinase-2 and Ras to treat melanoma (11). Besides, using GA to treat melanoma is associated with many benefits, such as high safety (12).

In recent years, significant inroads have led to the unprecedented finding that indoleamine 2,3-dioxygenase (IDO) inhibitors can reprogram the host inflammatory milieu to turn a “cold” tumor (non–T-cell inflammatory tumor) into a “hot” tumor (T-cell inflammatory tumor), becoming a research hotspot in the cancer immunotherapy field (13). An increasing body of evidence suggests that IDO is overexpressed in melanoma cells (14–16). As a small molecule inhibitor, IDO plays an important role in tumor drug resistance and immunosuppression (17, 18). The development and application of immune regulators have also been extensively studied. 1MT is a blocker of IDO that has been promoted and applied in clinical trials (19) and can inhibit tryptophan degradation to kynurenine and increase the antitumor effect of functional T cells (20, 21). Although IDO inhibitors are well tolerated, they yield a limited efficacy in cancer patients during clinical trials as monotherapy (22). Clinical trials have shown that, when combined with other drugs, it brings significant advantages over traditional drugs (23). Therefore, it is vital to explore combination therapy incorporating chemotherapy and immunotherapy to fight cancer.

**Scheme 1:**
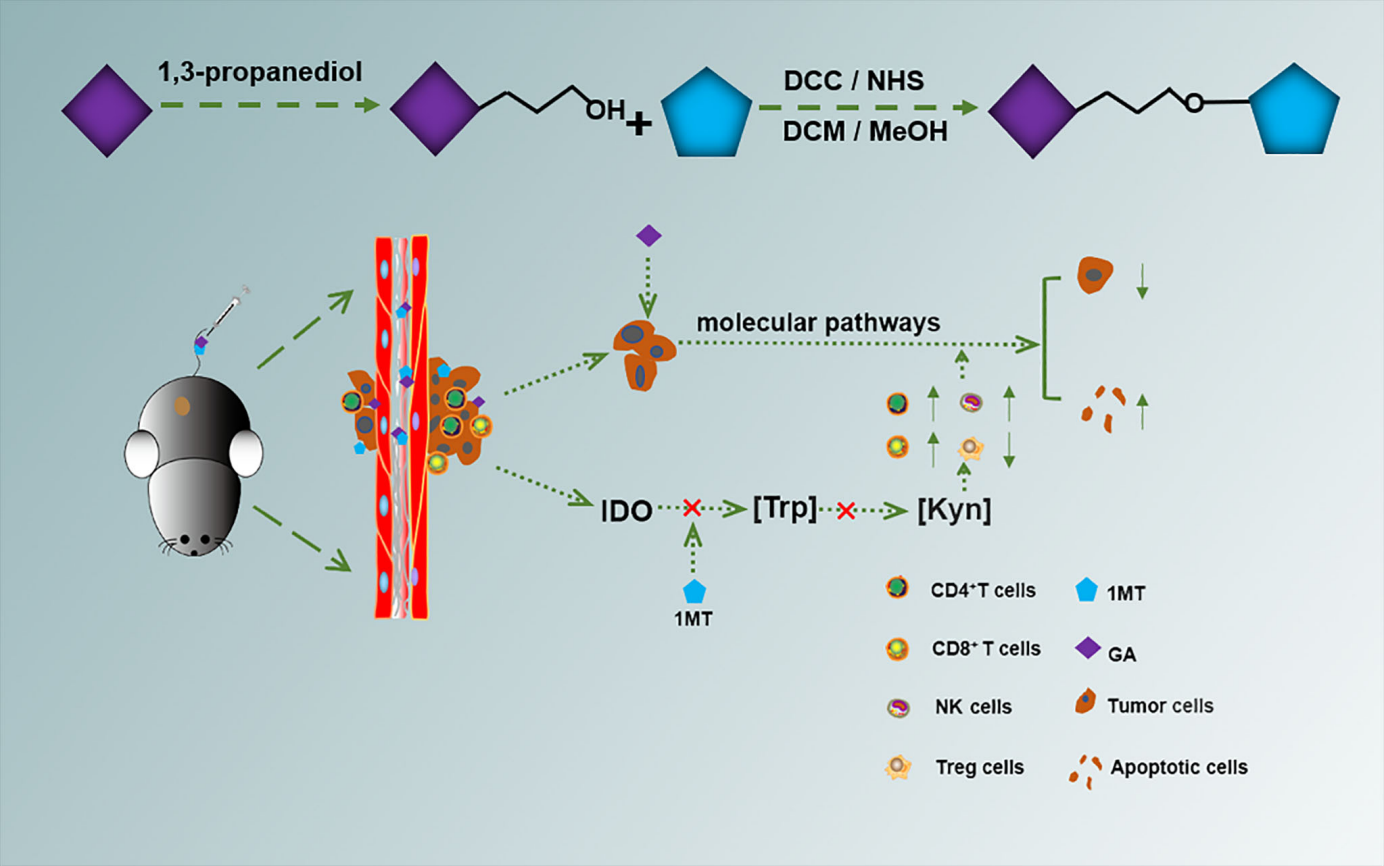
Schematic illustration for the preparation and the anticancer mechanism of GM.

Herein, we conjugated GA with 1MT through an ester bond to form a small molecule drug GA-1MT (GM). The hydrophobic drug 1MT was wrapped by the hydrophilic drug GA *via* ester bonds, improving the solubility of 1MT. (**Figure S1**). This strategy combines different drugs and improves solubility (**Scheme 1**). We substantiated that GM effectively controls melanoma growth through vivo and vitro experiments. Our findings suggest that GM has huge prospects for treating melanoma.

## Materials and Methods

### Materials

1,3-propanediol, p-toluenesulfonic acid, ethyl acetate, and sodium sulfate (Na2SO4), as well as dicyclohexylcarbodiimide (DCC), *n*-hydroxysuccinimide (NHS), and dimethylformamide (DMF) were purchased from Beijing Qiangsheng Technology Co., Ltd. 1MT was obtained from Sigma-Aldrich. GA and cell proliferation EdU image kits were purchased from Dalian Meilun Biotechnology Co., Ltd. The Annexin V-FITC/PI cell apoptosis detection kit was purchased from KeyGEN BioTECH and the Cell Counting Kit-8 (CCK-8) was from Beyotime Biotechnology. The radio immunoprecipitation assay lysis buffer, bicinchoninic acid protein assay kit, tyrosinase, proliferating cell nuclear antigen (PCNA) rabbit monoclonal antibody, cleaved caspase-3 rabbit monoclonal antibody, and horseradish peroxidase (HRP)–conjugated affinity Goat anti-rabbit/mouse immunoglobulin G (IgG) (H+L) were purchased from Beyotime Biotechnology. Matrigel was purchased from BD Biosciences, USA. Fluorescein isothiocyanate anti-mouse CD3 antibody, phycoerythrin (PE) anti-mouse CD8 antibody, antigen-presenting cell (APC) anti-mouse CD4 antibody, PE/Cy7 anti-mouse CD25 antibody, PE anti-mouse Forkhead box protein P3 (FOXP3) antibody, and APC anti-mouse natural killer (NK) 1.1 antibody were obtained from BioLegend Inc.

### Cell Culture

Murine melanoma cells (B16F10) were purchased from the Shanghai cell bank of the Chinese Academy of Sciences. B16F10 cells were cultured in Dulbecco’s modified Eagle’s medium (DMEM) medium containing 10% fetal bovine serum (FBS) and maintained in an incubator at 37°C in a humidified atmosphere consisting of 5% CO_2_.

### Animals

Female C57BL/6J mice aged 6–8 weeks were purchased from Beijing Vital River Laboratory Animal Technology Co., Ltd. The animals used in the experiment were cared for and used in accordance with the principles of experimental animal care and guidelines for the care and use of experimental animals. All animal experiments carried out were approved by the experimental animal ethics committee of Xuzhou Medical University.

### Preparation of GM

To prepare the small GM molecule, GA was dissolved with 1,3-propanediol, the organic layer was dried with Na2SO4, then the solvent was removed. DCC and NHS were added to DMF in batches, then distilled water was slowly added to the solution. Subsequently, it was dissolved in dichloromethane; trifluoroacetic acid was added slowly and stirred at room temperature for 4 h; the eluent was DCM: MeOH. The small chemical molecule GM was detected by 1HNMR and MS.

### *In Vitro* Cytotoxicity

B16F10 cells were seeded onto 96-well plates at a density of 6 × 10^3^ cells per well and inoculated with different concentrations of GM (0~250 μg/ml) overnight. Then, the cells were transferred to the CCK-8 solution for 4 h, and the cell viability was assessed by measuring sample absorbance at 450 nm by a microplate reader.

### EdU Cell Proliferation Assay *In Vitro*

B16F10 were seeded in 96-well plates at a density of 7 × 10^3^ cells per well and cultured in an incubator for 24 h. Then, phosphate-buffered saline (PBS), 1MT, GA, G + M, or GM (1MT: 7 μg/ml, GA: 200 μg/ml) were added and incubated for 48 h. First of all, according to the standard experimental method of EdU, diluted reagent A was added and incubated for 2 h, then fixed and washed with PBS containing 0.5% glycine Triton X-100, then reagents B, C, D, E, and F were added according to the standard steps. The photos were taken under a fluorescence microscope.

### FCM Analysis of Cells Apoptosis

B16F10 (3 × 10^5^ cells per well) were seeded in six-well plates and cultured for 24 h. Then, the adherent cells were incubated with PBS, 1MT, GA, G + M, and GM (1MT: 7 μg/ml, GA: 200 μg/ml), respectively, for 48 h. After being washed with cold PBS, they were collected and dyed with Annexin V-FITC/PI assay kit (KeyGEN BioTECH, China).Then, the FACSCalibur flow cytometer (BD Biosciences) was used to analyze cell apoptosis.

### Wound Healing Assay

B16F10 cells were seeded on six-well plates at a density of 3 × 10^5^ cells per well and incubated in a 5% CO2 humidified incubator at 37°C. Then, cells were scratched with a 10-μl pipette tip to create a wound, and the wound was formed along the scratch. The medium was discarded, and cells were washed carefully with PBS; the serum-free medium containing 1MT, GA, GA + 1MT, and GM (1MT: 7 μg/ml, GA: 150 μg/ml) was added and cells were incubated in an incubator for 24 and 48 h. The wound was observed, and pictures of cell migration were taken at 24 and 48 h by an Olympus microscope. Then, the images were analyzed and processed by ImageJ software at 0, 24, and 48 h of cell migration in the same field of view, and the migration area of the experimental cells was calculated.

### Transwell Cell Invasion Assay

Cell invasion was measured by a transwell migration assay with different treatments, including 1MT, GA, GA + 1MT, and GM (1MT: 32 μM, GA:1 50 μg/ml).For the invasion assay, the chamber was coated with Matrigel (BD Biosciences), the differently treated cells were seeded into the upper chamber with serum-free medium (2 × 10^5^ cells), and the bottom of the chamber contained the DMEM medium with 10% FBS. Following 48-h incubation, the cells on the upper surface of the polycarbonate films were gently removed with wet cotton swabs. The polycarbonate films were carefully removed from the upper chambers, and the cells in methanol were stained with 0.1% crystal violet. The cells were counted in five randomly selected fields of view with three replicates and photographed under an inverted microscope.

### Western Blot Analysis

PBS, 1MT, GA, G + M, and GM (1MT: 7 μg/ml, GA: 200 μg/ml) were added, and the mixture was incubated for 48 h. Cell lysates were collected for Western blot. After electrophoresis, the separated proteins were transferred onto a polyvinylidene difluoride membrane; the membrane was incubated in diluted primary antibody at 4°C overnight, including 1:200 mouse anti-tyrosinase (Santa Cruz Biotechnology, CA), PCNA (1:1000; catalog no.13110), and cleaved caspase-3 (1: 500; catalog no.13110). After incubation with the second antibody HRP-conjugated affinity Goat anti-rabbit/mouse IgG (H + L), the results were detected by enhanced chemiluminescence Western blotting kit. A chemiluminescence imaging system was used to quantify the intensity of the strip.

### *In vivo* Antitumor Experiments

All animal experiments were approved by the experimental animal ethics committee of Xuzhou Medical University. The animals used in the experiment were cared for and used in accordance with the “principles of experimental animal care” and “guidelines for the care and use of experimental animals”. A total of 1 × 10^6^ B16F10 cells were inoculated subcutaneously into the right side of groin of 6- to 8-week-old black female C57BL/6J mice to establish an *in situ* tumor model. The tumor volume was calculated according to the formula V = L× W2/2 (where L is the longest tumor diameter and W is the shortest tumor diameter). The tumors were allowed to grow to ~100 mm^3^ (7 days) before initiation of treatment. B16F10 primary tumor-bearing mice were randomly divided into five groups: (1) PBS, (2) 1MT, (3) GA, (4) GA + 1MT, and (5) GM (n = 11). Each group received intravenous injections of the corresponding drug every 2 days on days 0, 2, and 4. The doses of GA and 1MT were the same in each group: GA = 25 mg/kg, 1MT = 1.5 mg/kg. The tumor-bearing mice received intravenous injections every 2 days. The tumor volume and weight of the mice were measured every 2 days for 16 days. The mice were sacrificed on day 16, and the tumors were removed, weighed, and stained with immunofluorescence. The important organs were stained with hematoxylin and eosin (H&E). The spleen tissue was used for immunoassay.

### H&E, TUNEL, Ki67, and Immunofluorescence Assays

The mice were euthanized after 16 days of treatment. The liver, heart, kidney, spleen, lung, and tumor were collected for fixation, embedding, and sectioning. The paraffin-embedded tumor sections were dewaxed, hydrated, and antigen-repaired; the membrane was lysed and dripped with an appropriate amount of TdT-mediated dUTP nick-end labeling (TUNEL) and Ki67 staining solution to cover the tissue in the circle. 4',6-diamidino-2-phenylindole (DAPI) staining was used to re-stain the nuclei, then washed, and sealed. Then, the images were observed and collected with a fluorescence microscope.

### CD4 and CD8 Immunofluorescence

After 16 days of treatment, five mice in each group were killed, and tumor tissues were stripped and fixed in a 10% formaldehyde solution. The sections were dewaxed, hydrated, antigen-repaired, serum-sealed, and incubated overnight with primary antibody CD4 (1:500) or CD8 (1:200) at 4°C, then incubated with secondary antibody in the dark for 50 min. DAPI was used to re-stain the nucleus, quench the autofluorescence of the tissue, and seal the film. The images were observed and collected under a fluorescence microscope.

### Flow Cytometry

After 16 days of treatment, the spleens of mice in each group were removed. After grinding, the tissues were centrifuged for 5 min at 4°C, then 1 ml of red blood cell lysis buffer was added for lysis, washed with PBS, centrifuged, and the cells were resuspended at the concentration of 1 × 10^7^/ml. Then, according to the kit instructions, CD4 (catalog no.100412), CD8 (catalog no.100708), CD3 (catalog no.100204), and NK1.1 (catalog no.108706) antibodies were added to the cell suspension and incubated in the dark for 30 min. T_reg_ cells were detected with regulatory T-cell membrane breaking kit (catalog no.424401), CD4 (catalog no.100412), CD25 (catalog no.101916), and Foxp3 (catalog no.126404), consistent with the manufacturer’s instructions. The fluorescence intensity of cells was measured by flow cytometry (EMD Millipore).

### Statistical Analysis

All the experiments were made in triplicate, and the experimental values were expressed as the means ± standard deviation of three independent experiments. SPSS software 21.0 was used for statistical analysis. Overall survival was analyzed using the Kaplan–Meier method. A *p*-value of 0.05 was selected as the significance level, and the data were marked with (*) for p < 0.05, (**) for p < 0.01, and (***) for p < 0.001.

## Results

### Synthesis of GM

In this study, we synthesized a GM molecule to integrate different drugs for treating melanoma. The synthesis route of GM is shown in **Figure S1**. The structure of the synthesized PPS60 was verified by nuclear magnetic resonance ^1^H NMR and mass spectrum (**Figures S2** and **S3**). ^1^H NMR (400 MHz, DMSO-*d*_6_) was carried out to characterize the GM: δ 1.77–1.85 (m, 2H, -CH_2_-), 2.87–2.98 (m, 3H, -CH_2_-, -CH-), 3.66 (s, 3H, -NCH_3_), 4.04 (t, J = 6.0 Hz, 4H, -OCH_2_-, -CH_2_O-), 6.93 (s, 2H, Ar-H), 6.96 (d, J = 7.5 Hz, 1H, Ar-H). 7.04 (s, 1H, Ar-H), 7.07 (d, J = 7.4 Hz, 1H, Ar-H), 7.31 (d, J = 8.2 Hz, 1H, Ar-H), and 7.45 (d, J = 7.8 Hz, 1H, Ar-H). The mass and molecular formula of GM were determined to be MS (m/z): 429.10 [M + H]^+^; the mass calculated for C_22_H_24_N_2_O_7_ was 428.44, and that found was 428.10. These data verified that GM had been synthesized.

### GM Inhibited the Proliferation of B16F10 Cells *In Vitro*

**Figure 1:**
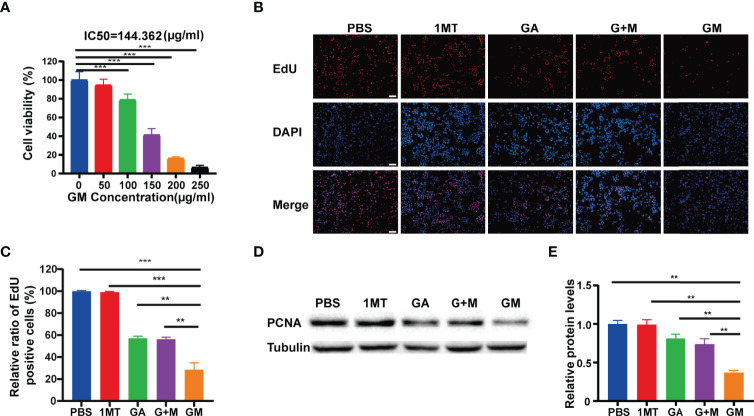
GA-1MT (GM) inhibited the proliferation of B16F10 cells *in vitro*. **(A)** Cell viability of B16F10 cells after culture with various concentrations. **(B)** Fluorescence images of B16F10 cells stained with EdU (red) and DAPI (blue) after different treatments (scale bar: 100 µm). **(C)** Quantitative analysis of EdU-positive cells after each treatment. **(D)** Western blotting to indicate the expression levels of PCNA in B16F10 cells from different groups. **(E)** Quantitative analysis of relative protein levels after various treatments. Data are presented as means ± SD, n = 3, **p < 0.01, ***p < 0.001.

In this study, the growth of B16F10 cells with different concentrations of GM was observed, and the survival rate of B16F10 cells was detected by CCK-8 assay. As shown in **Figure 1A**, when the GM concentration was 150 μg/ml, the survival rate of B16F10 cells was 38.49%. Interestingly, GM showed significant concentration-dependent cytotoxicity towards B16F10 cells. The results showed that the half-maximal inhibitory concentration (IC_50_) was 144.3 μg/ml (**Figure 1A**). To further study the effect of GM on the proliferation of B16F10 melanoma cells, we investigated the effect of PBS, 1MT, GA, G +M, and GM (GA: 200 μg/ml, 1MT: 7 μg/ml) on the proliferation of B16F10 cells by EdU analysis. As shown in **Figures 1B, C**, the proliferation rates of GA, G + M, and GM cells decreased to 57.2%, 56.3%, and 28.6%, respectively. The Western blot assay was used to further verify the effect of GM on the proliferation of melanoma cells. As shown in **Figures 1D, E**, PCNA protein level was significantly lower than in the GA and G + M groups, consistent with the EdU results. Compared to the GA group, administration of the mixture of GA and 1MT did not further decrease the proliferation of B16F10 cells and the expression of PCNA protein. However, the B16F10 cells receiving equivalent doses of GM exhibited significant growth inhibition compared to the G +M group. The results indicated that the inhibition effect of GM on the proliferation of B16F10 cells was more significant than single and combined treatments.

### GM Induced the Apoptosis of B16F10 Cells *In Vitro*

**Figure 2:**
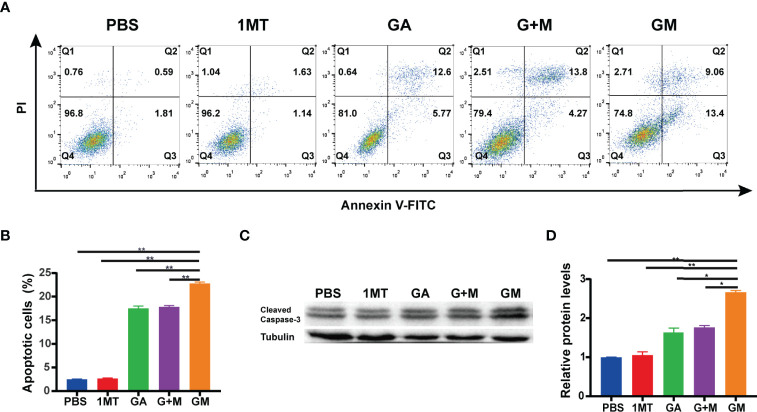
GM induced the apoptosis of B16F10 cells *in vitro*. **(A)** The apoptosis ratio was measured using Annexin V-FITC/PI apoptosis detection kit in B16F10 cells after the different treatments and the quantitative analysis **(B)**. **(C)** The expression of cleaved caspase-3 was detected by Western blot with different drugs, and their average gray values were quantified **(D)**. Data are presented as means ± SD, n = 3, *p < 0.05, **p < 0.01.

There is ample evidence suggesting that inhibited apoptosis can promote the growth of tumor cells. Flow cytometry was used to detect the apoptosis of melanoma cells of B16F10 cells incubated with different drugs for 48 h. As displayed in **Figures 2A, B**, compared with the control group, the early and late apoptosis rates of B16F10 cells after incubation with GA, G + M, and GM increased to 18.37%, 18.33%, and 22.46%, respectively, corroborating that GM could significantly promote the apoptosis of melanoma B16F10 cells. We conducted Western blotting to further verify the promotion of apoptosis. As shown in **Figures 2C, D**, Western blot showed that the expression of cleaved caspase-3 was significantly higher than GA and G + M treatment groups after 48 h, which was consistent with the flow cytometry results.

In addition, inhibition of melanin production can reportedly improve the therapeutic effect of melanoma (24, 25). It is well established that tyrosinase is a key enzyme that regulates melanin production. We assessed changes in tyrosinase protein expression *in vitro*. In this study, Alpha-melanocyte stimulating hormone (α-MSH) was used to promote melanin cell proliferation *in vitro* for 5 days. At the same time, different drugs were added. As revealed in **Figure S4**, compared with the α-MSH stimulation group, the tyrosinase protein expression level in the GM group decreased in a dose-dependent manner and was lower than in the GA group at a GA concentration of 25 μg/ml. Therefore, GM can inhibit tyrosinase protein levels in a dose-dependent manner.

### GM Inhibited B16F10 Cell Invasion and Migration

**Figure 3:**
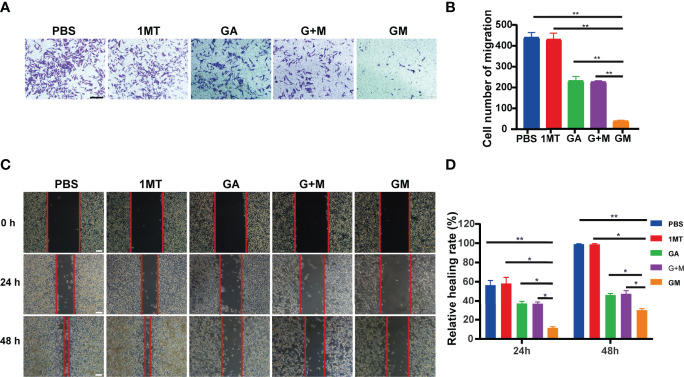
GM inhibited B16F10 cell invasion and migration. **(A)** Invasion of B16F10 cells was analyzed using the transwell assay after diverse treatments (scale bar: 200 µm). **(B)** Corresponding normalized number of cells that penetrated through the membrane. **(C)** Images of wound healing experiments after diverse treatments. (Scale bar: 100 µm). **(D)** Quantitative analysis of wound healing assay. Data are presented as means ± SD, n=3, *p < 0.05, **p < 0.01.

We further assessed the role of GM in the invasion of B16F10 melanoma. The effects of different drugs on B16F10 cell invasion after culture for 48 h were detected by the transwell assay. **Figures 3A, B** showed that tumor cell invasion in the GM group was significantly lower than in the single-and combined-drug groups. In conclusion, GM significantly inhibited the invasion of B16F10 cells. The metastatic ability of tumor cells is reportedly closely related to the prognosis and survival of patients. Accordingly, we carried out wound healing experiments to analyze the effect of GM on cell migration. As presented in **Figures 3C, D**, the wound healing assay showed that the inhibitory effect of GM on migration was stronger than in the single-and combined-drug groups.

### GM Yields a Significant Anti-Melanoma Effect *In Vivo*

**Figure 4:**
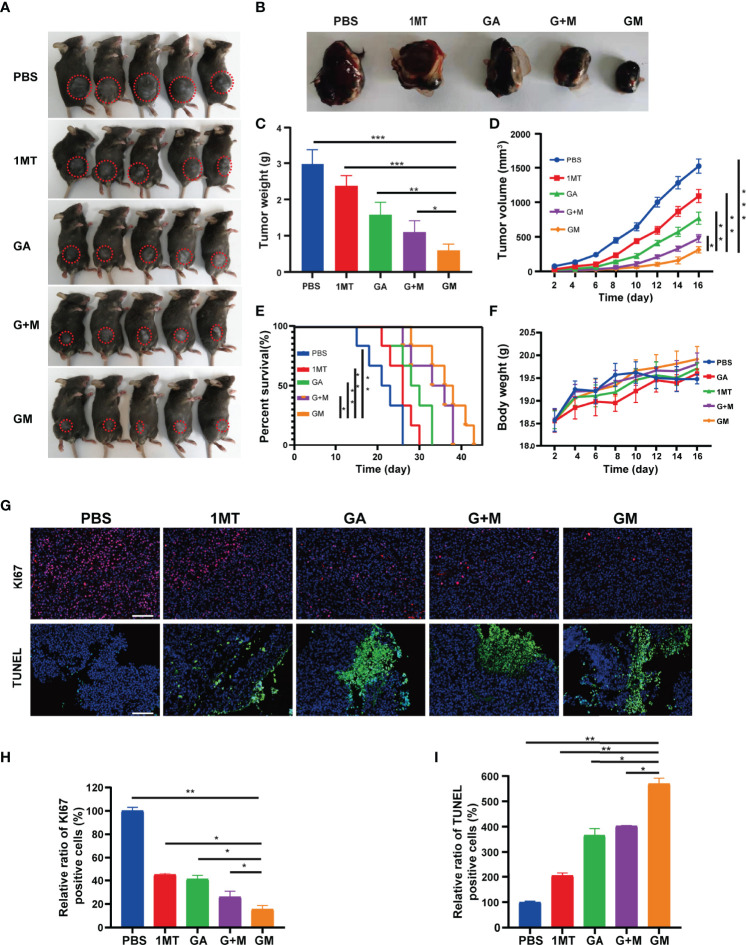
Antitumor effect evaluation in the B16F10 cell tumor model. **(A)** Representative digital images of mice under different treatments (tumor: red circle). **(B)** Digital images of tumor tissues removed from mice after different treatments. **(C)** Tumor weight and **(D)** tumor growth curve of the tumor-bearing mice from different treatments. **(E)** Survival curve and **(F)** bodyweight of the tumor-bearing mice from different treatments. **(G)** Representative immunofluorescence images of Ki67 and TUNEL staining of tumor tissues after the corresponding treatments (scale bar: 200 µm). **(H, I)** Quantitative analysis of KI67 and TUNEL staining of tumor tissues from different groups. Data are presented as means ± SD, *p < 0.05, **p < 0.01, ***p < 0.001.

Based on the remarkable treatment efficacy of GM *in vitro*, we further investigated its *in vivo* anticancer effect using a B16F10 orthotopic tumor-bearing mouse model. The mice were randomly separated into five groups (11 mice per group) for different treatments: group 1: PBS; group 2: 1MT; group 3: GA; group 4: GA + 1MT; group 5: GM. Overall, tumor-bearing mice in group 5 yielded the smallest red circles, which represented the size of the tumor (**Figure 4A**). The tumor size and body weight were measured every 2 days after treatment, and the mice were euthanized on day 16 for *in situ* inspection of tumor weight. The tumors were collected to visually display the renderings of the resected tumors (**Figure 4B**), the mean tumor weight (**Figure 4C**), and the mean tumor volume (**Figure 4D**). We found that the tumor size in groups 1 and 2 rapidly increased, whereas groups 3 and 4 exhibited a slight tumor inhibition effect. Most importantly, group 5 mice showed a satisfactory tumor-suppressing effect, yielding the lowest tumor weight (**Figure 4C**) and the smallest tumor volume (**Figure 4D**) after 16 days of treatment. In addition, the survival curve in **Figure 4E** showed that the survival rate of GM treatment was higher than with 1MT, GA alone, and combination treatment. **Figure 4F** indicated no significant weight changes in the mice in different groups. Subsequently, Ki‐67 expression exhibited that the GM group caused significantly lower cell proliferation than other groups (**Figures 4G, H**). Moreover, the expression of TUNEL was further investigated to evaluate the tumor cell apoptosis. As expected, tumor cells in the GM group showed significantly higher expression than in other groups (**Figures 4G, I**). These results confirmed that GM yielded a better tumor suppression performance than alternative approaches (1MT or GM or 1MT + GM). Besides, as shown in **Figure S5**, there was no obvious major organ injury after various treatments, indicating the safety of GM for tumor treatment.

### GM-Mediated Immune Response in the B16F10 Cell Tumor Model

**Figure 5:**
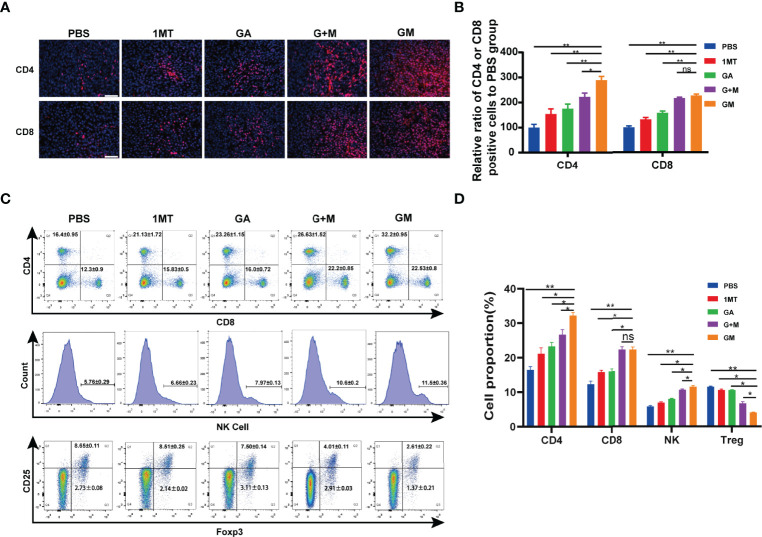
GM-mediated immune response in the B16F10 cell tumor model. **(A)** Representative immunofluorescence images of the tumor showing CD4^+^ T cell and CD8^+^ T cell after the corresponding treatments and **(B)** the quantitative analysis (scale bar: 200 µm). **(C)** The ratios of CD4^+^, CD8^+^, NK1.1, and CD4^+^Foxp3^+^ T cells in the spleen were detected by flow cytometry after the corresponding treatments and **(D)** the quantitative analysis. Data are presented as means ± SD, ns, no significance, *p < 0.05, **p < 0.01.

To assess whether GM is effective against the immune system, immunofluorescence analysis was performed on tumor tissue samples. CD4 and CD8 staining methods were used to evaluate the effect of GM on T-cell infiltration in tumor tissues *in vivo*. The results are shown in **Figures 5A, B**. CD4^+^ T-cell staining and CD8^+^ T-cell staining were almost negative in the control group, indicating almost no T-cell infiltration. The red fluorescence area (CD4^+^ T and CD8^+^ T) of the 1MT and GA groups was larger than that in the control group, designating that the single-drug group exhibited increased infiltration of T cells in tumor tissue. The infiltration area of red fluorescent regions of CD4^+^ T cells and CD8^+^ T cells in the two groups was increased compared with the single-drug and control groups, and more T cells were infiltrated. *In vitro* experiments proved that the combined treatment could eloquently improve clinical efficacy. In contrast with the combined treatment group, GM caused significantly increased CD4^+^ T cells but not CD8^+^ T cells. However, the interaction between ester bonds and the cell membrane can activate the drug for cell internalization, thus increasing the infiltration of immune cells in the tumor by inhibiting IDO in the immune microenvironment. Consistently, it has been shown that the immune effect of GM on melanoma is enhanced by promoting the reaction of CD4^+^ T and CD8^+^ T lymphocytes.

The spleen is the center of immune cell aggregation. To analyze whether different drugs can promote the increase of tumor-infiltrating T cells, we examined the proportion of CD4^+^ and CD8^+^ T cells, NK1.1, and CD4^+^Foxp3^+^ T_reg_ in the spleen of mice. As shown in **Figures 5C, D**, at the end of GM treatment, the proportion of adaptive immune cells CD4^+^, CD8^+^ T cells, and innate immune cells, including NK1.1 cells, increased remarkably compared with other groups, whereas T_reg_ cells (such as CD4^+^Foxp3^+^) decreased. Moreover, the proportion of T cells was significantly increased, and the number of T_reg_ cells decreased compared with other groups (**Figure 5C**). These results indicated that GM could synergistically regulate the immune response through GA and 1MT and improve the antitumor effect.

## Discussion

Current evidence suggests that melanoma has strong immunogenicity (26, 27). Reports of drug resistance to traditional drugs have increased in recent years, mediated by immune escape, limiting treatment efficiency and shortening the annual median survival (13, 28). Indeed, much emphasis has been placed on identifying low-cost effective drugs with minimal side effects. Over the years, immunotherapy has represented an innovative and promising strategy for treating solid tumors by evoking antitumor immunity (29). Overwhelming evidence substantiates that IDO can be overexpressed in various tumor cells, which plays an important role in tumor drug resistance and immunosuppression (19, 30). Clinical studies have shown that blocking IDO can have positive antitumor effects by overcoming the related immune resistance and enhancing tumor immunotherapy (31). With the rapid development of IDO inhibitors, 1MT has been used in clinical trials as a blocker of IDO (19). Notwithstanding that IDO inhibitor monotherapy demonstrated tumor inhibition potential, the inhibition ability remains limited. Importantly, clinical trials have shown that IDO brings many advantages over traditional drugs when combined with other drugs (4).

Based on the combination of immunosuppressants approach in clinical trials, we propose the combination therapy strategy of natural polyphenol GA and 1MT. It is widely acknowledged that natural polyphenols can be extracted from various plants, which has the advantages of traditional medicine, including low cost and high safety (32). The most encouraging thing is that, in recent years, studies have uncovered that natural polyphenols can inhibit the progress of tumors *via* a variety of mechanisms, for example, decreasing of PD-L1 expression through binding to EGFR in Non-small-cell lung cancer (33). Through cell cycle regulation, MAPK pathway, and p13k-akt pathway, it can inhibit tumor proliferation, induce apoptosis, and influence tumor progression through epigenetic modification (34–36). In conclusion, it has been confirmed that natural polyphenols have multitarget antitumor behavior, and the drug resistance rate is significantly reduced. Overall, combination therapy represents a novel approach to improve the current clinical treatment approach.

At the same time, we also stand on the basis of previous studies, the tumor acid environment, ester bond breaking, and drug intelligent release. This is also due to our efforts to overcome the resistance of traditional chemotherapy drugs (37). In the present study, we synthesized a hitherto undocumented GM molecule by the chemical reaction of carboxyl group and alcohol group linked by ester bonds from GA and 1MT yielding a single small molecule depending on the acid environment of the tumor. Due to the presence of ester bonds, the hydrophobic drug (1MT) can be encapsulated by the hydrophilic drug (GA), which improves the drug solubility (37). The CCK-8 test showed that GM (100–250 g/ml) inhibited the growth of B16F10 melanoma cells, whereas 1MT was limited by solubility and immune microenvironment and did not produce significant toxicity against B16F10 cells *in vitro* (37). We then evaluated the effect of GM on proliferation (**Figure 1**) and expression of the apoptotic proteins (**Figure 2**) of melanoma cells *in vitro*. Nuclear protein PCNA plays a key role in cell proliferation during the G1-M phase of the cell cycle and interacts with multiple chaperones in DNA repair and methylation (38). It has been found that natural polyphenols can induce apoptosis in various tumor cells (38, 39). In this study, cleaved caspase 3 and PCNA protein expression was upregulated, which confirmed that GM significantly inhibited the proliferation of tumor cells and induced apoptosis of B16F10 cells. Melanoma is a tumor with high metastasis and invasion potential with a high mortality rate (40, 41). Importantly, we substantiated that GM treatment could inhibit the migration and invasion of B16 cells (**Figure 3**).

Studies have found that melanin production can produce a relatively hypoxic environment due to increased oxygen consumption, making the tumor resistant to traditional drugs and photodynamic therapy. Therefore, inhibition of melanin production can improve the therapeutic effect against melanoma (24, 25). Moreover, we found that GM can inhibit the expression of tyrosinase in a dose-dependent manner to achieve a more efficient synergistic treatment of melanoma (**Figure S4**). As an inhibitor of IDO, 1MT has been extensively studied in the research of immune regulators. This combination therapy strategy has greatly improved the antitumor efficacy (42, 43). Therefore, a novel molecule consisting of NL8189 and GA integrated by ester bond formation produced an improved antitumor effect.

In this study, we found that GM can be used to treat tumors *in situ in vivo* (**Figure 4**). The results showed that, compared with PBS or other single treatment groups and combined treatment groups, the tumor growth of the GM group was significantly inhibited, and the survival rate was prolonged. NK cells and adaptive immune cells (such as helper CD4^+^ T cells and memory CD8^+^ T cells) play an important role in the antitumor immune response (44). In this study, after GM treatment, flow cytometry (**Figure 5**) showed that CD4^+^ T and CD8^+^ T cells and NK1.1 cells increased in the spleen. Tumor immunofluorescence showed that CD4^+^ T and CD8^+^ T cells were also increased in the tumor, and the innate immune and adaptive immune responses remained consistent, interacting to achieve an effective antitumor effect. As previously mentioned, Foxp3 is an important member of the forehead transcription factor family. Its expression and function are closely related to T_reg_ cells (45). T_regs_ play key roles in inducing immunosuppression of cancer cells, which mobilize the immune microenvironment and inhibit cytotoxic cells allowing cancer cells to escape immune attacks (46–48). Unexpectedly, the GM group exhibited a more significant decrease in the number of T_reg_ cells.

In conclusion, these results suggest that our chemically synthesized small molecule drug GM has huge prospects for clinical application for immunotherapy in melanoma patients since it is safe and effective.

## Data Availability Statement

All datasets generated for this study are included in the article/**Supplementary Files**. Further inquiries can be directed to the corresponding author.

## Ethics Statement

The use of animals was approved by the experimental animal ethics committee of Xuzhou Medical University.

## Author Contributions

GJ and HML conceived the research design and supervised data analysis. CC drafted the manuscript. HG, WYJ and DLX performed the experiments and analyzed the data. All authors read and approved the final manuscript.

## Funding

This work was supported by the National Natural Science Foundation of China [No. 81872493], the Jiangsu Provincial Medical Talent Foundation, the ‘Six Talent Peaks’ Project of Jiangsu Province (No. WSW-074), Social Development Project of Jiangsu Department of Science and Technology [No. BE2020647].

## Conflict of Interest

The authors declare that the research was conducted in the absence of any commercial or financial relationships that could be construed as a potential conflict of interest.

## Publisher’s Note

All claims expressed in this article are solely those of the authors and do not necessarily represent those of their affiliated organizations, or those of the publisher, the editors and the reviewers. Any product that may be evaluated in this article, or claim that may be made by its manufacturer, is not guaranteed or endorsed by the publisher.
