# Supplementary material for: IDO Inhibitor and Gallic Acid Cross-Linked Small Molecule Drug Synergistic Treatment of Melanoma
Source: Front Oncol. 2022 Jul 1;12:904229. doi: 10.3389/fonc.2022.904229 (PMC9303008; doi:10.3389/fonc.2022.904229)
Supplement: Supplementary file 1 [file DataSheet_1.docx]

Supplementary Material

# Methods and Materials synthesis

# Synthesis of GA-1,3-propanediol (compound a)

Add gallic acid and 1,3-propanediol into the reaction bottle and stir evenly; Then add p-toluenesulfonic acid and raise the temperature to 120 ℃; After 5 hours of reaction, pour into a large amount of cold water and add ethyl acetate for extraction; Wash the organic layer with water three times, dry the organic layer with anhydrous Na2SO4 and spin off the solvent; The crude product was purified by silica gel column and eluted with eluent to obtain compound a.

# Synthesis of GA-1,3-Propanediol-Boc-D-Trp (Me)-OH (compound b)

Compounds Boc-D-Trp (Me) - OH, DCC and NHS were added to DMF in batches; After stirring at room temperature for 1 hour, add compound ga-1,3-propanediol and continue stirring for 10 hours; After the reaction, water and ethyl acetate were added for extraction, and the organic layer was dried with anhydrous Na2SO4 and the solvent was removed; The crude product was purified by silica gel column and eluted to obtain compound b.Synthesis of GM (compound c)

# Supplementary Figures and Tables

Dissolve compound b in DCM, slowly add TFA, stir at room temperature for 4 hours, unscrew DCM, add water (insoluble, add a few drops of methanol); Adjust pH to 7-8 with NaOH aqueous solution; DCM was added, the organic layer was dried with anhydrous Na2SO4 and the solvent was removed; The crude product was purified by silica gel column and washed with DCM and MeOH to obtain the final product GM.

The chemical small molecule GM was detected by ^1^H NMR, and the specific synthetic products were detected by ^1^H NMR:^1^H NMR (400 MHz, D2O) δ 7.73 (D, j = 7.9 Hz, 1H), 7.51 (D, j = 7.9 Hz, 1H),7.34 (s, 2h),7.21 (D, J = 8.4 Hz, 2h),4.39 (s, 2h),4.06 (s, 1H),3.79 (D, j = 12.6 Hz, 3H),3.36 (s, 2h), 2.75 (s, 2h),2.02 (s, 3H).

**Supplementary Figure 1.** Synthetic route of GM

2. Characterization of GM-3

^1^H NMR (400 MHz, DMSO-d6) δ 1.77-1.85 (m, 2H, -CH2-)，2.87-2.98 (m, 3H, -CH2-,-CH-), 3.66 (s, 3H, -NCH3), 4.04 (t, J = 6.0 Hz, 4H, -OCH2-,-CH2O-), 6.93 (s, 2H, Ar-H), 6.96 (d, J = 7.5 Hz, 1H，Ar-H). 7.04 (s, 1H, Ar-H), 7.07 (d, J = 7.4 Hz, 1H, Ar-H), 7.31 (d, J = 8.2 Hz, 1H, Ar-H), 7.45 (d, J = 7.8 Hz, 1H, Ar-H).MS (ESI) m/z [M-H]+: 429.16, found: 429.1，[M-H]-: 427.16, found: 427.1。


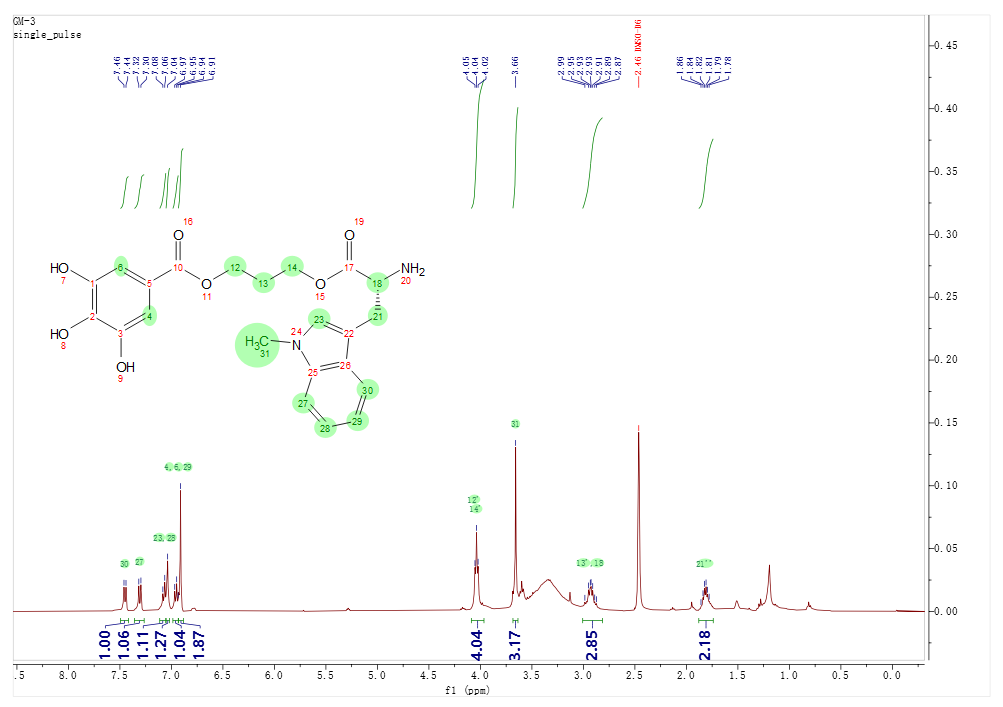


**Supplementary Figure 2.** ^1^H NMR spectra of compound GM-3


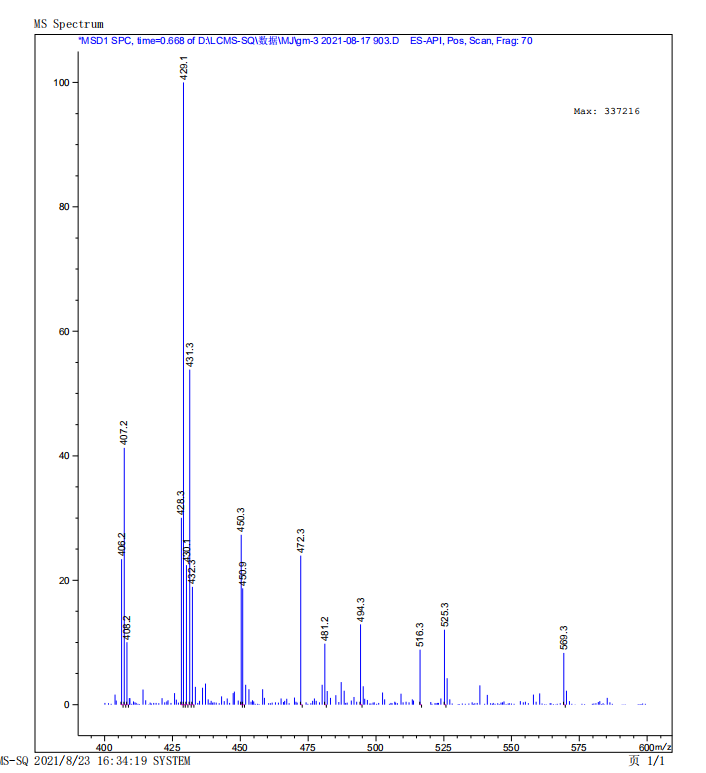


**Supplementary Figure 3.** MS spectra of compound GM-3

**
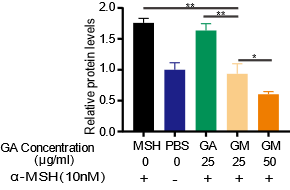

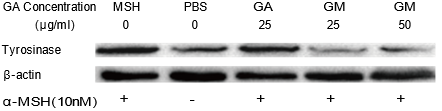
3. Results**

**Supplementary Figure 4.** After treated with GA and GM for 5 days, and incubated with or without α-MSH (10nm) for stimulation, The level of protein tyrosinase expression was detected by western blotting.


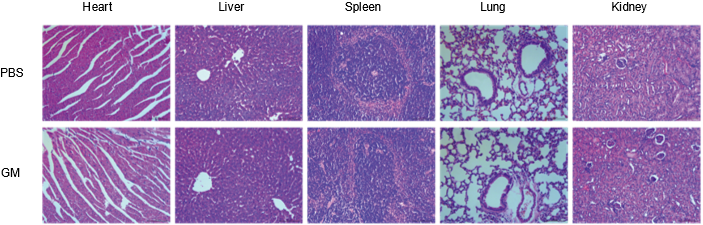


**Supplementary Figure 5.** Systemic toxicity study of GM. Liver, H&E staining in sections from major organs after mice treated with GM.
